# Mindfulness-Based Stress Reduction, Fear Conditioning, and The Uncinate Fasciculus: A Pilot Study

**DOI:** 10.3389/fnbeh.2016.00124

**Published:** 2016-06-15

**Authors:** Britta K. Hölzel, Vincent Brunsch, Tim Gard, Douglas N. Greve, Kathrin Koch, Christian Sorg, Sara W. Lazar, Mohammed R. Milad

**Affiliations:** ^1^Department of Neuroradiology, Klinikum Rechts der Isar, Technical University of MunichMunich, Germany; ^2^Department of Psychiatry, Massachusetts General Hospital, Harvard Medical SchoolBoston, MA, USA; ^3^Institute for Complementary and Integrative Medicine, University Hospital Zurich and University ZurichZurich, Switzerland; ^4^Translational Neuromodeling Unit (TNU), Institute for Biomedical Engineering, University of Zurich and Swiss Federal Institute of Technology (ETH)Zurich, Switzerland

**Keywords:** mindfulness, meditation, fear conditioning, fear extinction, diffusion tensor imaging, uncinate fasciculus, neuroplasticity

## Abstract

Mindfulness has been suggested to impact emotional learning, but research on these processes is scarce. The classical fear conditioning/extinction/extinction retention paradigm is a well-known method for assessing emotional learning. The present study tested the impact of mindfulness training on fear conditioning and extinction memory and further investigated whether changes in white matter fiber tracts might support such changes. The uncinate fasciculus (UNC) was of particular interest in the context of emotional learning. In this pilot study, 46 healthy participants were quasi-randomized to a Mindfulness-Based Stress Reduction (MBSR, *N* = 23) or waitlist control (*N* = 23) group and underwent a two-day fear conditioning, extinction learning, and extinction memory protocol before and after the course or control period. Skin conductance response (SCR) data served to measure the physiological response during conditioning and extinction memory phases. Diffusion tensor imaging (DTI) data were analyzed with probabilistic tractography and analyzed for changes of fractional anisotropy in the UNC. During conditioning, participants were able to maintain a differential response to conditioned vs. not conditioned stimuli following the MBSR course (i.e., higher sensitivity to the conditioned stimuli), while controls dropped the response. Extinction memory results were not interpretable due to baseline differences. MBSR participants showed a significant increase in fractional anisotropy in the UNC, while controls did not (group by time interaction missed significance). Pre-post changes in UNC were correlated with changes in the response to the conditioned stimuli. The findings suggest effects of mindfulness practice on the maintenance of sensitivity of emotional responses and suggest underlying neural plasticity. (ClinicalTrials.gov, Identifier NCT01320969, https://clinicaltrials.gov/ct2/show/NCT01320969).

## Introduction

Mindfulness meditation originally stems from ancient Asian traditions (Bhikkhu, 2008), and has been adapted for Western contexts in the form of stress reduction programs to promote health and enhance stress resilience (Kabat-Zinn, 1990). Mindfulness is defined as the awareness that emerges through deliberately and non-judgmentally paying attention to present moment experiences, such as emotions, body sensations, and thoughts (Kabat-Zinn, 1990). While numerous reports have documented the beneficial effects of mindfulness practice on variables of mental and physical health (Keng et al., 2011; Gotink et al., 2015), the working mechanisms of mindfulness meditation are poorly understood (Vago and Silbersweig, 2012; Lutz et al., 2015; Tang et al., 2015). One of the mechanisms that appears crucial in the process of promoting mental health is the modification of emotional responses (Grecucci et al., 2015).

Through mindfulness training, practitioners learn to encounter emotional experiences with a distinct internal attitude. They let themselves be affected by whatever experience is present in the field of awareness, refraining from engaging in avoidance or internal reactivity towards it, and instead bring acceptance to bodily and affective responses (Hart, 1987). Practitioners are instructed to meet even unpleasant emotions by turning towards them, rather than turning away (Santorelli, 2000).

Several theoretical accounts have argued that through this particular internal attitude (i.e., exposure to emotional experiences without reactivity), mindfulness practice might influence basic conditioning and/or extinction processes (Baer, 2003; Brown et al., 2007; Hölzel et al., 2011b; Vago and Silbersweig, 2012; Brewer et al., 2013; Tang et al., 2015). Such processes are typically investigated through classical fear conditioning and extinction protocols. Fear conditioning is a learning process in which a neutral stimulus [such as a light or a tone, conditioned stimulus (CS)] is paired with an aversive unconditioned stimulus (US, such as an aversive electric shock). After a few pairings, the presentation of the CS comes to elicit various conditioned fear responses, such as enhanced skin conductance response (SCR) in humans. Repeated presentations of the CS in the absence of the US result in the extinction of all conditioned responses. Extinction is thought to form a new memory trace (Rescorla, 2001; Quirk, 2002), or reconsolidate the old memory with new contextual associations (Nader and Einarsson, 2010; Rossato et al., 2010; Inda et al., 2011); extinction memory is thought to compete with conditioned memory for control of fear expression (Myers and Davis, 2007). Recent research has shown that successful extinction memory reliably differentiates healthy from pathological conditions (Milad et al., 2008; Holt et al., 2009; Graham and Milad, 2011). Extinction memory may thus be a critical process in the transformation of maladaptive states.

The conceptual similarity of mindfulness with an exposure situation might suggest that mindfulness meditation could enhance extinction memory processes. Through enhanced attentional skills and non-judgmental awareness, it is also conceivable that mindfulness impacts the process of learning of emotional responses, i.e., that it impacts the conditioning of emotional responses (Brewer et al., 2013). To our knowledge, no research has been published to date that has empirically tested such claims.

Studies that have examined mindfulness meditation-induced structural and functional changes in the brain have identified a number of implicated brain areas (Hölzel et al., 2011b; Fox et al., 2014; Tang et al., 2015), including limbic regions (e.g., Hölzel et al., 2010, 2011a), and regions in the orbitofrontal cortex (e.g., Hölzel et al., 2008). These brain regions have been identified as critical for the acquisition as well as the extinction of conditioned fear responses, and for the retention of fear extinction memory (Milad et al., 2005, 2007; Sehlmeyer et al., 2009; Milad and Quirk, 2012). More broadly, the interplay between prefrontal regions and the limbic system supports the regulation of emotions (Banks et al., 2007).

It has previously been shown in cross-sectional research that in meditators compared to controls, structural connectivity is pronounced within multiple white matter pathways throughout the entire brain, including the uncinate fasciculus (UNC; Luders et al., 2011). It has also been demonstrated in one longitudinal study that changes can occur in the integrity of white matter tracts in the brain following a relatively brief meditation training Integrative Body Mind Training (IBMT; Tang et al., 2010). While the described neuroplastic changes are intriguing and might potentially underlie the benefits ascribed to the practice of mindfulness meditation, it has often been neglected to actually test the relationship between neuroplastic processes and changes in behavior. The UNC is the long-range association fiber that connects the orbito-frontal cortex with limbic structures within the anterior temporal lobe (Von Der Heide et al., 2013). While its exact function is currently not clear (Von Der Heide et al., 2013), it is likely that it is involved in processes of emotion regulation and emotional learning, based on the brain regions that it connects (Kim et al., 2011).

We conducted a pilot quasi-randomized controlled longitudinal study to investigate the impact of an 8-week Mindfulness-Based Stress Reduction (MBSR) course on the physiological response (SCR) in an emotional learning paradigm, i.e., a 2-day fear conditioning, extinction learning, and extinction retention experiment. We hypothesized that participation in the MBSR course would lead to facilitated extinction memory and also investigated the impact on the conditioning phase. The study further tested for plasticity of white matter integrity in the UNC, and investigated a potential relationship between changes in structural connectivity and changes in emotional learning. In order to ensure that the MBSR course had the typical beneficial effects on well-being in participants, self-report measures of perceived stress, mindfulness, and difficulties in emotion regulation (DER) were included.

## Materials and Methods

### Participants

**Figure 1:**
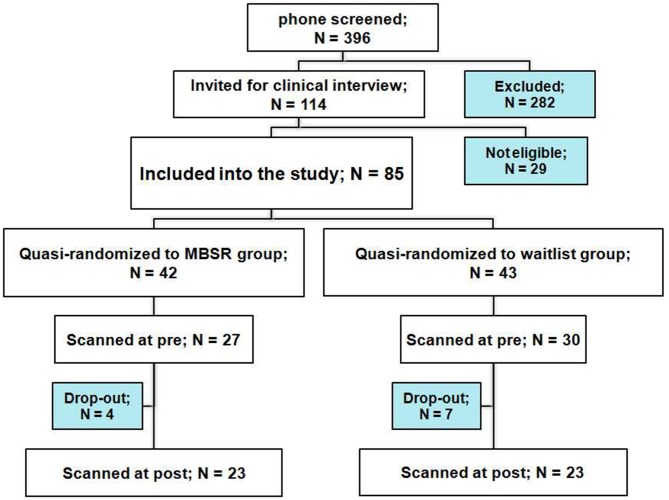
**Participant flow chart**.

Participants were recruited from the Boston area (MA, USA) through newspaper and online advertisements, seeking volunteers that were feeling stressed. For a complete participant flow chart see Figure 1. Following a phone screen and the Structured Clinical Interview for DSM-IV Disorders MINI version (First et al., 2002), 85 volunteers were included in the study. Participants were eligible if they were 18–65 years of age, proficient in English, right-handed as self-reported and reported at least a moderately high stress level [score of ≥3 on the 4-item Perceived Stress Scale (PSS)]. Exclusion criteria were: diagnosis of a psychiatric or neurological disorder, significant previous meditation or yoga experience (≥10 lifetime meditation or yoga classes, and ≥5 within the last year), medication that affects cerebral metabolism, major or chronic medical condition, history of head injury, seizures or stroke, as well as the usual MR imaging safety exclusion criteria.

Twenty-three participants in each group completed the pre- and post-testing. Individuals in the waitlist group took the MBSR course after completing all testing. Sample characteristics are reported in Table 1. The two groups differed in mean age, and a difference in the number of days between the pre and post scans showed a trend-significance; therefore, “age” and “days between scans” were included as covariates in all analyses.

**Table 1:** **Sample characteristics (N, age, gender, years of education and days between scans) for the mindfulness-based stress reduction (MBSR) and control groups and statistics for the group comparison**.

	MBSR	Waitlist control	Statistics of group comparison
*N*	23	23	
Age (SD)	35.0 (10.0)	28.9 (8.0)	*T*_(44)_ = −2.31; *p* = 0.026
Gender	Female: 12	Female: 17	*χ*^2^_(1)_ = 2.33; *p* = 0.22
Years of education (SD)	17.7 (1.7)	17.1 (2.0)	*T*_(44)_ = −1.19; *p* = 0.24
Days between scans (SD)	68.8 (13.1)	77.1 (18.7)	*T*_(44)_ = 1.75; *p* = 0.09

Participants provided written informed consent prior to enrollment into the study. The protocol was approved by the Partners Healthcare Institutional Review Board and the study was conducted according to the principles expressed in the Declaration of Helsinki. Participants received $200 compensation for completing all testing, and received the MBSR course for free.

### Procedure

All participants needed to agree to be randomized to either of the conditions; and were allocated to the respective experimental group based on the time of their enrollment into the study (i.e., quasi-randomization). Within 2 weeks before their participation in the MBSR course or respective waitlist period (“pre” testing visit), participants underwent a 2-day fear conditioning, extinction, and extinction memory protocol in the MRI scanner during which SCR data was collected. Before beginning MRI scanning, participants chose the level of the electric stimulation to be “highly annoying but not painful.” Diffusion tensor imaging (DTI) data and self-report questionnaire data were also collected at that time. fMR imaging was also performed, but will be reported elsewhere. Participants completed the exact same testing procedure within 3 weeks following the MBSR course, and within a similar time interval in the control group (“post”).

### Intervention: Mindfulness-Based Stress Reduction Course

The 8-week MBSR program consisted of weekly group meetings lasting two and a half hours each, plus one full day (6.5 h) during the 6th week of the course. The program is described extensively elsewhere (Kabat-Zinn, 1990). All classes were taught by a senior teacher from the Center for Mindfulness at the University of Massachusetts Medical School. Participants received audio recordings containing 45-min guided mindfulness exercises for daily homework practice, and recorded the amount of time they spent engaged in mindfulness exercises each day.

### Questionnaires

A battery of self-report questionnaires were administered at the pre and post testing timepoint via the REDCap online system^1^, including three that were relevant for the present study:

The PSS (Cohen and Williamson, 1988) is a 14-item, validated, and widely used instrument for assessing the degree to which situations in the subject’s life are perceived as stressful, including questions related to how unpredictable or uncontrollable those events are perceived to be.

The Difficulties in Emotion Regulation Scale (DERS) has 36 items and contains six factors: (a) non-acceptance of emotional responses (nonaccept); (b) difficulties engaging in goal-directed behavior (goals); (c) impulse control difficulties (impulse); (d) lack of emotional awareness (aware); (e) limited access to emotion regulation strategies (strategies); and (f) lack of emotional clarity (clarity). It has high internal consistency, good test-retest reliability, and adequate construct and predictive validity (Gratz and Roemer, 2004). Since we didn’t have hypotheses regarding the six factors, a single score (sum of all items) was used for the present study (effects of the intervention on scores of the subscales are reported for completeness).

The Mindful Attention Awareness Scale (MAAS; Brown and Ryan, 2003) is focused on the presence or absence of attention to and awareness of what is occurring in the present and consists of 15 items. It has been shown to hold good internal consistency (Cronbach’s *α* = 0.82). It is the most widely cited instrument to measure trait mindfulness and its psychometric properties are supported by a larger number of studies than for any other instrument (Park et al., 2013). For a critical discussion of content validity of mindfulness questionnaires see Grossman (2008).

### Fear Conditioning, Extinction, and Extinction Retention Protocol

Before and after the MBSR course or wait period, all subjects underwent a 2-day fear conditioning protocol, that was carried out in an MRI scanner, and that was identical to that as previously reported (Milad et al., 2005, 2006, 2007, 2008). Differential Aversive Conditioning Software (DAVCOND) controlled and timed the monitor with the images for the protocol and the finger stimulator while recording SCR-data simultaneously. Visual stimuli were rear-projected on a screen at the head of the scanner and could be seen by the participant through an adjustable mirror mounted to the head coil.

In the Conditioning phase (day 1), two CS, e.g., a red and a blue light, were paired with the US (an electric stimulation, selected by participants to be highly annoying but not painful) at a partial reinforcement rate of 62.5%. One CS was extinguished during the subsequent extinction phase on day 1 (CS+ E), whereas the other was not (CS+ U). A third CS (e.g., a yellow light) was presented during the conditioning phase and never paired with the US (CS−). The US was delivered immediately following CS+ offset, with no delay between the CS+ offset and the US onset. The extinction learning phase began after approximately 1 min following the conditioning phase. On day 2, CS+ E, CS+ U, and CS− were presented during the recall phase and no electric stimulation was delivered.

In accordance with previously published procedures (Orr et al., 2000), recording electrodes were attached to the palm of the subject’s left hand to measure SCR, and stimulating electrodes were connected to two fingers of the subject’s right hand through which electric stimulation was delivered. Subjects were not asked to meditate during the protocol, since we were interested in the effects of mindfulness meditation practice on everyday behavior.

#### Skin Conductance Response Data

SCR was measured through a 9-mm (sensor diameter) Sensor Medics Ag/AgCl electrodes. As previously described (Milad et al., 2007, 2009), SCR for each stimulus was calculated by subtracting the mean level for the 2 s immediately preceding stimulus onset from the maximum value recorded during the stimulus interval. Thus, SCRs to the stimuli reflect changes in SCR level beyond any change in SC level produced by the context or drift over time. For the present study, data were median-filtered.

The following parameters were obtained in order to test our research questions:

1. *Enhanced extinction memory*: the average response to the first two CS+ E trials of the extinction recall phase (day 2) is divided by the largest response to a CS+ E trial during the conditioning phase (day 1; the square root of each value is used) and then multiplied by 100, yielding a percentage of maximal conditioned responding; which is subtracted from 100% to yield the extinction retention index (ERI; cf., Zeidan et al., 2012).
2. *Differential conditioning*: the mean values of the response to the CS− trials were subtracted from the mean of the response to CS+ E and CS+ U trials during the conditioning phase (excluding the first presentation of each stimulus, as well as the first CS− after the switch between the CS+ E and CS+ U). Additional analyses on the CS+ and CS− variables separately were included as follow up analyses.

Matlab Version R2012a, The MathWorks, Inc., Natick, MA, USA, was used for SCR data processing.

Due to a number of technical problems, SCR data was only available for a subset of subjects. Data for a subject was included if all of the following criteria were met: (a) data of the phase(s) of interest were recorded for both the “pre” and “post” testing visit, and did not contain any obvious problems or distortions (data of two participants in the MBSR group and one in the control group were lost); (b) subjects exhibited a reaction to the US during the conditioning phase at both visits (defined as ≥0.05 increase in SCR following the US; three MBSR and two control participants were excluded); (c) subjects exhibited conditioned responses during the conditioning phase at both visits (defined as ≥0.05 increase in SCR following the CS+; another four participants in the MBSR and seven participants in the control group were excluded).

### Diffusion Tensor Imaging Data

#### Data Acquisition

Data was collected on a Siemens Magnetom Trio 3.0 Tesla whole body high-speed imaging device with an 32-channel gradient head coil. Head movement was restricted using foam cushions.

Diffusion-weighted images were acquired using a conventional 2D spin-echo echo-planar imaging (EPI) sequence. The series included 60 images acquired with diffusion weighting along non-collinear directions (*b* = 700 s/mm^2^) and 10 images without diffusion weighting (*b* = 0 s/mm^2^). The isotropic resolution was 1.9 mm with 72 axial slices and no gap between them. The image matrix size was 112 × 112, echo time (TE) = 87 ms and repetition time (TR) = 9040 ms. Furthermore, the flip angle was 90°, dimensions of the field-of-view (FOV) in the 2D-images were both 213 mm, the GRAPPA acceleration factor was two and the bandwidth was 1440 Hz/Px.

T1-weighted images were acquired using a 3D multi-echo magnetization-prepared rapid acquisition with gradient echo (MEMPRAGE) sequence, including motion correction and re-acquisition of slices corrupted by motion (Tisdall et al., 2012). Slice thickness was 1 mm with image matrix size 256 × 256 × 176. The four TEs were 1.74, 3.6, 5.46 and 7.32 ms, TR was 2530 ms and inversion time TI was 1340 ms, flip angle was 7°, FOVs were both 256 mm, GRAPPA acceleration factor was two, and all bandwidths were 651 Hz/Px. The echo spacing was 9.6 ms and the final output considered for analysis was computed as the root-mean-squared combination of the four echoes.

#### Data Analysis

TRACULA (TRActs Constrained by UnderLying Anatomy) is a method for automated reconstruction of a pre-defined set of major white-matter pathways using a combination of diffusion-weighted MR images and anatomical constraints. TRACULA is based on the global probabilistic approach (Jbabdi et al., 2007) and utilizes prior anatomical information from Freesurfer analyses (Yendiki et al., 2011). DTI data processing was performed using the standard procedures for TRACULA software. Preprocessing and reconstruction of the T1 anatomical data was done using the FreeSurfer longitudinal stream^2^ Software package version 5.3, as this procedure is unbiased by the number of time points and more robust when multiple time points are available (Reuter et al., 2012). Preprocessing of the DTI data included image corrections for eddy currents and simple head motions with FSL’s eddy correct, B0 distortion correction using the field map data, and computation of the diffusion tensor. The affine intra-subject registration with the T1-weighted images was done using Freesurfer’s “bbregister” (Greve and Fischl, 2009). Inter-subject registration was done using the affine inter-subject registration to MNI152 space. The TRACULA standard procedures were then performed to fit the ball-and-stick model of diffusion to the diffusion-weighted images and for probabilistic tracking and track segmentation. For each tract, TRACULA computes a summary FA measure by averaging the FA in each voxel along the tract, where each voxel is weighted by the likelihood that the voxel was in the given tract. We examined the effects of mindfulness training on this measure of the UNC FA to test our study hypothesis. For completeness we also tested the other tracts that were not part of our primary hypothesis.

### Statistical Analysis

Effects of condition and time on the dependent variables were assessed by conducting repeated measures analysis of variance (rmANOVA), with time as repeated measure, and group as between-subjects factor. Age and the number of days between the two scan sessions were entered as covariates. Paired samples *t*-tests were conducted to compare pre and post timepoint scores within each group, univariate analyses of variance tested for differences between the groups at the pre scan, and Pearson correlations assessed correlations between variables. SPSS 20.0 was used for data analysis.

## Results

### Amount of Mindfulness Home Practice

Participants in the MBSR group attended an average of 7.35 classes and all but three participants attended the all-day retreat. The complete set of home practice logs was available for eleven participants, and twelve did not complete all logs; a mean of 5.85 out of 7 home practice logs was available. Missing logs were replaced with averaged practice time for the individual. The average practice time across the entire course was 25.14 h (SD: 11.56; min: 4.12; max: 44.67), equal to an average of 30 min per day.

### Questionnaires

Data were available for all participants except for one in the control group, who only completed the PSS. Table 2 shows the detailed statistics. The group by time interaction was significant for all questionnaire total scores (age and days between scans included as covariates). In regard to DERS subscales, the group by time interaction was significant for “goals” and “strategies”, and showed a trend significance for “nonaccept”. Follow-up paired *t*-tests within the MBSR group showed a significant increase for the total scores of the DERS and MAAS, and the DERS subscales “nonaccept”, and “aware”. Baseline differences were present for the DERS and MAAS total scores, but were not significant. The DERS aware scale was the only to show a significant group difference. Questionnaire total scores were highly correlated at baseline: PSS-DERS: *r* = 0.67, *p* < 0.001; PSS-MAAS: *r* = −0.45, *p* = 0.002; DERS-MAAS: *r* = −0.77, *p* < 0.001 (partial correlations, controlling for age and days between scans), demonstrating that they do not assess separate constructs.

**Table 2:** **Results of the statistics for the questionnaire data: (a) the interaction of the 2 × 2 (group by time) rmANOVA; (b) values at pre and post for the MBSR group and results of a paired-samples *t*-test; (c) values at pre and post for the control group and results of a paired-samples *t*-test; (d) baseline differences between the MBSR and control groups**.

Questionnaire	Interaction*	MBSR pre	MBSR post	Change	Controls pre; mean (SD)	Controls post; mean (SD)	Change	Baseline differences*
PSS	*F*_(1,42)_ = 4.28; *p* = 0.045	22.65 (8.19)	21.04 (5.56)	*T*_(22)_ = 1.25; *p* = 0.23	20.87 (9.61)	23.26 (7.88)	*T*_(22)_ = −1.60; *p* = 0.12	*F*_(1,42)_ = 0.41; *p* = 0.53
DERS total	*F*_(1,41)_ = 6.70; *p* = 0.013	79.65 (19.27)	71.13 (20.89)	*T*_(22)_ = 2.46; *p* = 0.02	70.27 (19.34)	74.95 (22.15)	*T*_(21)_ = −1.53; *p* = 0.14	*F*_(1,41)_ = 2.08; *p* = 0.16
DERS nonaccept	*F*_(1,41)_ = 3.80; *p* = 0.058	12.13 (4.84)	10.17 (5.04)	*T*_(22)_ = 2.32; *p* = 0.03	10.32 (4.14)	10.64 (4.40)	*T*_(21)_ = −0.59; *p* = 0.56	*F*_(1,41)_ = 1.24; *p* = 0.27
DERS goals	*F*_(1,41)_ = 6.13; *p* = 0.02	14.57 (4.43)	13.48 (5.20)	*T*_(22)_ = 1.54; *p* = 0.14	13.00 (4.44)	14.45 (5.20)	*T*_(21)_ = −1.78; *p* = 0.09	*F*_(1,41)_ = 1.59; *p* = 0.21
DERS impulse	*F*_(1,41)_ = 0.35; *p* = 0.56	10.17 (3.01)	9.70 (4.04)	*T*_(22)_ = 0.74; *p* = 0.47	10.36 (5.00)	10.64 (4.58)	*T*_(21)_ = −0.42; *p* = 0.68	*F*_(1,41)_ = 0.10; *p* = 0.76
DERS strategies	*F*_(1,41)_ = 8.49; *p* = 0.01	15.52 (6.54)	13.70 (5.73)	*T*_(22)_ = 1.59; *p* = 0.13	13.68 (5.52)	15.82 (6.77)	*T*_(21)_ = −2.40; *p* = 0.03	*F*_(1,41)_ = 2.08; *p* = 0.16
DERS aware	*F*_(1,41)_ = 2.42; *p* = 0.13	16.74 (4.53)	14.30 (4.14)	*T*_(22)_ = 2.49; *p* = 0.02	13.45 (4.63)	13.50 (5.15)	*T*_(21)_ = −0.07; *p* = 0.94	*F*_(1,41)_ = 3.04; *p* = 0.09
DERS clarity	*F*_(1,41)_ = 1.13; *p* = 0.29	10.52 (3.06)	9.78 (3.01)	*T*_(22)_ = 1.31; *p* = 0.21	9.45 (2.89)	9.91 (2.96)	*T*_(21)_ = −0.73; *p* = 0.47	*F*_(1,41)_ = 0.37; *p* = 0.55
MAAS	*F*_(1,41)_ = 7.44; *p* = 0.009	3.63 (1.03)	4.17 (0.85)	*T*_(22)_ = −3.292; *df* = 22; *p* = 0.003	4.08 (1.04)	4.00 (0.93)	*T*_(21)_ = 0.712; *p* = 0.48	*F*_(1,41)_ = 1.22; *p* = 0.28

*Age and days between scans were included as covariates.

**Figure 2:**
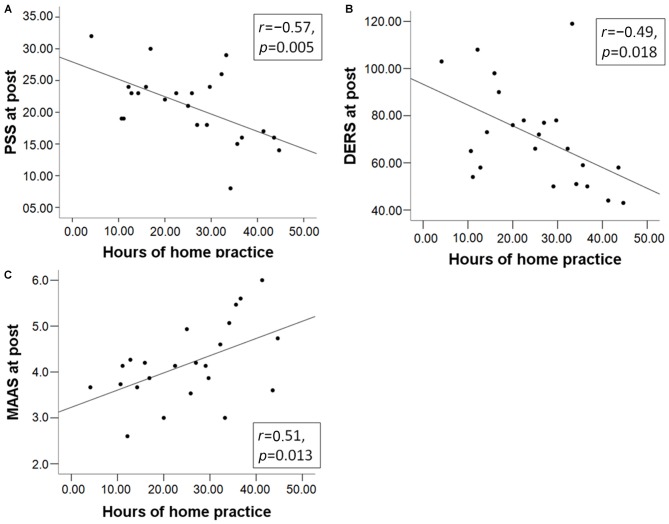
**The total number of hours of mindfulness home practice in the mindfulness-based stress reduction (MBSR) group predicts the total scores of (A) the Perceived Stress Scale (PSS); (B) Difficulties in Emotion Regulation Scale (DERS); and (C) Mindful Attention Awareness Scale (MAAS) at post**.

We also investigated whether the amount of home practice predicted questionnaire scores. Regression parameters were significant for all questionnaire scores at post (PSS: ß = −0.57, *t*_(22)_ = −3.17, *p* = 0.005; DERS: ß = −0.49, *t*_(22)_ = −2.57, *p* = 0.018; MAAS: ß = 0.51, *t*_(22)_ = 2.73, *p* = 0.013; Figure 2). The pre-post change in questionnaire scores was not correlated with the amount of home practice (all *p*-values ≥ 0.3). At pre, only the DERS (ß = −0.53, *t*_(22)_ = −2.83, *p* = 0.010) negatively predicted the amount of home practice, possibly indicating an impeding effect on engagement in home practice. Note that results are not corrected for multiple comparisons. Results from the analysis of questionnaire data suggest that MBSR course participants reported a beneficial effect on measures of mindfulness and emotion regulation capacities, and effects were stronger for those who engaged in more mindfulness home practice.

### SCR Data

There were no systematic effects in the selected levels of shocks between the visits and groups. Mean stimulation current (mA) for MBSR participants was 1.62 (SD: 0.62) at pre and 1.72 (SD: 0.81) at post. For controls, it was 1.50 (SD: 0.66) and 1.55 (SD: 0.61) respectively. There was no difference between groups at pre (*t*_(44)_ = 0.64, *p* = 0.53) or post (*t*_(44)_ = 0.80, *p* = 0.43), no pre post change within the MBSR (*p* = 0.27) or control (*p* = 0.43) groups and the group by time interaction was not significant (*F*_(1,42)_ = 0.15; *p* = 0.71).

#### Extinction Memory

For the investigation of extinction memory, data from 11 participants in the MBSR group and eight participants in the control group were usable. As predicted, the repeated measures ANOVA on the ERI showed a significant group by time interaction (*F*_(1,15)_ = 5.089, *p* = 0.039, *η*^2^ = 0.25). In the MBSR group, the ERI increased significantly from pre (*M* = 32.14, SD = 34.70) to post (*M* = 62.77, SD = 24.94; *t*_(10)_ = −2.41, *p* = 0.037), whereas it did not change in the control group from pre (*M* = 72.82, SD = 38.06) to post (*M* = 67.65, SD = 33.81; *t*_(7)_ = 1.06, *p* = 0.33). However, the difference between the groups at pre was significant (*t*_(17)_ = 2.42, *p* = 0.027). We therefore entered the pre ERI scores (along with age and days between scans) into an ANOVA with the change of ERI as the dependent variable. While the pre score significantly predicted the change of ERI (*F*_(1,14)_ = 9.39; *p* = 0.008, *η*^2^ = 0.40), the effect of the group was no longer significant (*F*_(1,14)_ = 0.41, *p* = 0.53). There were no significant correlations between the amount of homework practice or questionnaire scores with the change in the ERI.

#### Differential Conditioning

**Figure 3:**
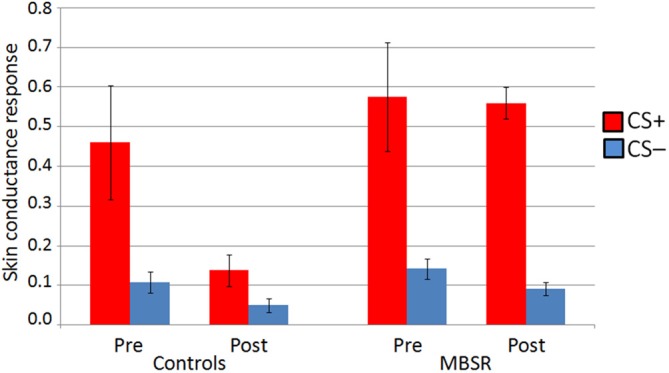
**Skin conductance response (SCR) to CS+ and CS− in the conditioning phase at pre and post in the MBSR and control groups.** Bars represent standard error of the mean.

Data from 14 participants in the MBSR group and 13 participants in the control group were usable for the analysis of SCR data in the conditioning phase. The repeated measures ANOVA revealed a significant interaction regarding the difference of the response to the CS+ vs. the CS− during the conditioning phase (*F*_(1,23)_ = 5.304, *p* = 0.031, *η*^2^ = 0.19; controlling for age and days between scans; Figure 3). A rmANOVA on the response to the CS+ alone also showed a significant interaction (*F*_(1,23)_ = 4.38, *p* = 0.048, *η*^2^ = 0.16), while the interaction was not significant for the response to the CS− (*F*_(1,23)_ = 0.068, *p* = 0.80). A paired samples *t*-test revealed that within the control group, there was a decrease in the response to the CS+ (pre *M* = 0.46 (SD: 0.52); post *M* = 0.14 (SD: 0.15); *t*_(12)_ = 2.34, *p* = 0.037). At pre, groups did not differ in the differential conditioning variable, nor in the responses to CS+ or CS− alone (*p* values ≥ 0.47). For an illustration see Figure 3.

**Figure 4:**
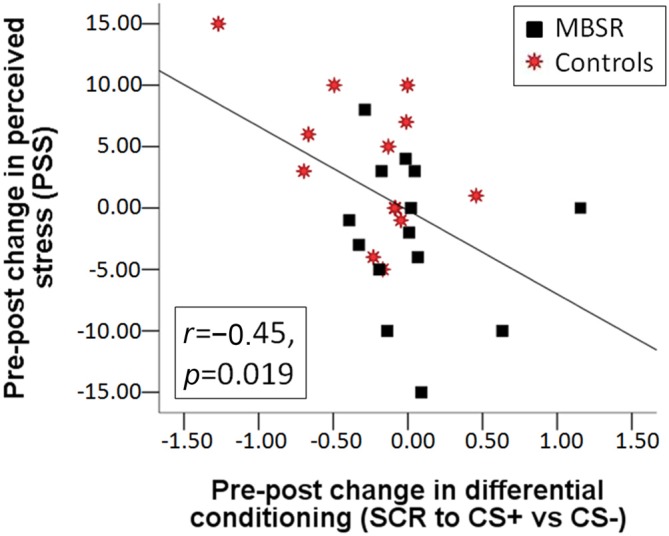
**Correlation between the change in perceived stress from pre to post and the change from pre to post in differential conditioning (the difference of the SCR response to CS+ vs. the response to CS−) across the MBSR (black square) and control (red star) groups**.

Change in the differential conditioning variable (diff CS+ vs. CS−) was not correlated with homework practice. The pre-post change in differential conditioning (diff CS+ vs. CS−) was correlated with the pre-post change in PSS (*r*_(25)_ = −0.447, *p* = 0.019) across both groups (Figure 4), as was the change in the response to the CS+ (*r*_(25)_ = −0.386, *p* = 0.047); this correlation was mostly driven by participants in the control group (*r*_(11)_ = −0.533 and *r*_(11)_ = −0.516). The more participants increased their perceived stress, the greater the decrease they showed in their differential response, and response to the CS+.

### DTI Data

**Figure 5:**
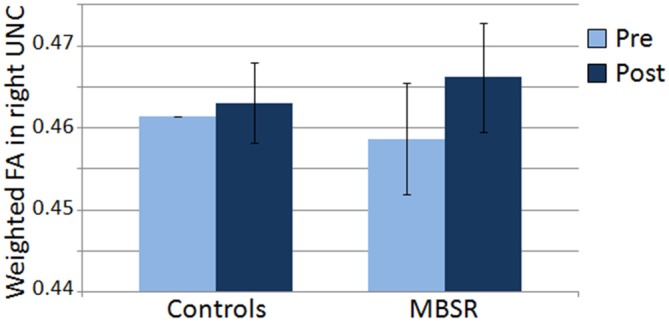
**Mean of the weighted averaged fractional anisotropy within the right uncinate fasciculus (UNC) in the control and MBSR groups before (pre) and after (post) the MBSR course or wait period respectively.** Bars represent standard error of the mean.

**Figure 6:**
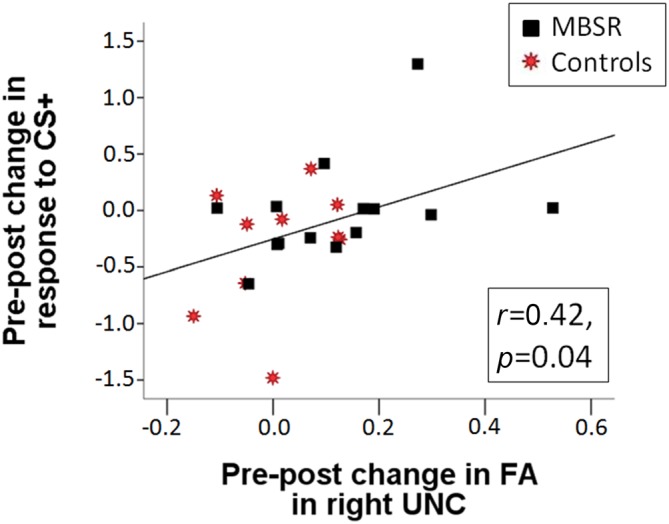
**The pre-post change in SCR in response to the CS+ is positively correlated with the pre-post change in fractional anisotropy (FA) in the right UNC across the MBSR (black square) and control (red star) groups (*r*_(22)_ = 0.422, *p* = 0.040)**.

DTI data was available for 21 participants in the MBSR group and 17 participants in the control group. Mean FA results for each group and time point for the right UNC are shown in Figure 5. Within the MBSR group, there was a significant increase in the weighted averaged FA signal for the right UNC from pre (*M* = 0.459, SD = 0.032) to post (*M* = 0.467, SD = 0.032; *t*_(20)_ = −2, 09, *p* = 0.049), while there was no change within the control group (pre *M* = 0.461, SD = 0.021, post *M* = 0.463, SD = 0.020; *t*_(16)_ = − 0.755, *p* = 0.46). However, rmANOVAs showed no significant interaction between group and time point for either the right UNC (*F*_(1,34)_ = 1.62, *p* = 0.21) or the left UNC (*F*_(1,34)_ = 0.001, *p* = 0.98). The change in weighted averaged FA signal from pre to post was correlated with the change in response to the CS+ (*r*_(22)_ = 0.42, *p* = 0.04; Figure 6), and with the change in differential conditioning from pre to post by *r* = 0.39, which showed a trend towards significance (*p* = 0.062, *df* = 22). FA values were not correlated with questionnaire scores or the amount of home practice.

No other tracts showed a change from pre to post in the MBSR group. In the control group, there was a significant change in the weighted averaged FA signal in the left cingulum—cingulate gyrus (CCG; pre *M* = 0.498, SD = 0.034; post *M* = 0.511, SD = 0.036; *t*_(16)_ = −3.429, *p* = 0.003) and a trend towards a change in the left superior longitudinal fasciculus temporal (SLFT; *t*_(16)_ = −2.06, *p* = 0.056). A significant group by time interaction was found for the left CCG (*F*_(1,35)_ = 5,88, *p* = 0.021; corrected for age and days between scans; all *p*-values are not corrected for multiple comparisons) and for the left SLFT (*F*_(1,35)_ = 5.591, *p* = 0.024). FA values were not correlated with questionnaire scores or SCR data.

## Discussion

This study investigated changes in fear conditioning and extinction, as well as changes of white matter integrity in the UNC following an 8-week MBSR course, compared to a waitlist control group. In regard to conditioning, MBSR participants were able to maintain the initial response to the conditioned stimuli, while controls dropped from pre to post. MBSR participants further showed an increase in FA in the UNC, while controls showed no change. The change in fractional anisotropy in the UNC was positively correlated with the SCR to the CS during the conditioning phase. Due to a relatively small sample size, this study only has the status of a pilot trial, and therefore findings are to be taken as suggestive.

### Effects on Self-Report Measures

Self-report questionnaires assessed effects on participants’ PSS, MAAS, and DERS. The MBSR group reported improvements in mindfulness and in DER, which were significantly stronger compared to the control group, where no changes occurred. These findings are in line with previous studies (Robins et al., 2012; Garland et al., 2013; Omidi and Zargar, 2014). While baseline differences between the control and MBSR groups were not statistically significant, small changes existed and might have worked in favor of our hypotheses through regression to mean effects. However, the present study also suggested that the amount of home practice in the MBSR group predicted scores of the three questionnaires at post, further corroborating the impact of the mindfulness practice on the self-report effects. These findings demonstrate that participants perceived beneficial effects from the MBSR course.

Interestingly, the DERS score at pre negatively predicted the amount of home practice. This finding suggests that difficulties in the capacity to regulate one’s emotions make it harder for participants to engage in mindfulness home practice. Mindfulness programs that have been developed for patient populations with particular DER address this problem by employing specific ways to support patients in taking up an individual home practice (Deckersbach et al., 2014).

### Effects on Fear Conditioning

Results demonstrated an effect of the MBSR course on the acquisition of conditioned fear. While the SCR to the CS+ (and consequently also the differential response) dropped significantly in the control group from pre to post, it remained high following the MBSR course. A previous study that had investigated test—retest reliability of responses in the fear conditioning, extinction, and extinction memory paradigm did not find significant changes over time (Zeidan et al., 2012), however, small decreases in SCR were noted for baseline values, response to the UCS, and responses to the CS+ during conditioning. This prior study did not relate changes in SCR to changes in stress. Higher SCR to the CS+ is indicative of successful fear learning and is adaptive for healthy functioning. In contrast, response to the CS+ during fear acquisition can be diminished during anxiety states (Vriends et al., 2011) or in dissociative states (Ebner-Priemer et al., 2009).

While we had no directed hypothesis regarding the effect on the acquisition of conditioned fear, a preservation of the sensitivity to the conditioning process makes sense in the light of what is being trained by mindfulness practice: mindfulness training teaches to remain open, non-judgmental, and curious to what is occurring from moment to moment and refrain from engaging in internal avoidance. Consequently, previous studies have reported decreased habituation in experienced meditation practitioners; i.e., they maintain the freshness of attention for each incoming stimulus (Kasamatsu and Hirai, 1966; Antonova et al., 2015). The literature, however, is mixed, and enhanced habituation has also been reported in beginning meditators relative to controls (Antonova et al., 2015).

Here, we found a correlation between the change in the reaction to the CS+ (and differential conditioning) and the change in perceived stress, i.e., remaining sensitive to the CS+ was associated with diminished stress. This finding further supports the idea that the preservation of the response to the CS+ is adaptive. As an alternative interpretation, maintained sensitivity to the CS+ might have been caused by enhanced study commitment in the MBSR group, who had more contact with lab personnel. Such an effect could equally influence questionnaire scores. This effect could be controlled through the inclusion of an active control group.

### Effects on Extinction Memory

Successful extinction memory differentiates healthy from pathological conditions (Milad et al., 2008; Holt et al., 2009; Graham and Milad, 2011). Extinction learning and its retention may thus be a critical process in the transformation of maladaptive states. It allows individuals to learn not to have a fear response to neutral stimuli when there is no adaptive function for the fear response. Therefore, fear extinction brings individuals in a position where they experience a sense of safety and can flexibly elicit other emotional and behavioral reactions. Based on conceptual considerations, as well as based on the overlap of involved brain regions, we have argued that mindfulness might work by enhancing extinction memory processes (Hölzel et al., 2011b; Tang et al., 2015) and have tested this hypothesis in the present study. While we found the expected group by time interaction and a significant increase in the MBSR group regarding extinction memory, the strong influence of baseline differences precludes the interpretation of this finding. Future research will need to employ larger sample sizes, and might consider stratifying participants to the groups based on baseline values of extinction retention.

### Change in Fractional Anisotropy in the Uncinate Fasciculus Following MBSR

Here, we investigated changes in fractional anisotropy in the UNC following the MBSR course and aimed to relate potential changes to the effects on behavioral parameters. Previous research had reported that experienced meditators show greater white matter integrity within multiple white matter pathways throughout the entire brain (Luders et al., 2011), and changes can occur following a relatively brief meditation training (Tang et al., 2010). These previous studies, however, did not test for the behavioral relevance of such modifications.

In the present study, we were particularly interested in the UNC, due to its potential role for emotion regulation and emotional learning. In a review of literature on the UNC, Von Der Heide et al. (2013) suggest that one role is to transmit salience-laden stimulus representations stored in anterior-medial aspects of the temporal lobe to the lateral orbital frontal cortex. The lateral orbital frontal cortex has been shown to be related to assigning rewards and punishments to behaviors, especially in the context of associative learning (Noonan et al., 2010). The role of the UNC might thus be to allow decisions to be made based on the emotional tone or incentive value of the stimuli in order to alter behavior—i.e., approaching or avoiding stimuli based on the reward and punishment history (Von Der Heide et al., 2013). This description of the role of the UNC is well in line with the present finding that UNC plasticity is related to effects on the differential response to a conditioned vs. not CS.

The present study revealed a significant increase in fractional anisotropy in the right UNC following MBSR, and no significant change in the control group. However, the group by time interaction missed significance, and data therefore can’t be taken to support the study hypothesis. Our sample sizes are relatively small though, so it will be worthwhile for future research to pursue this hypothesis further, and employ greater sample sizes. An impact of MBSR on UNC plasticity could have important implications, given that DTI studies have suggested that the structural integrity of this pathway is negatively correlated with trait anxiety levels (Kim and Whalen, 2009; Baur et al., 2012; Modi et al., 2013), subthreshold posttraumatic stress disorder and hyperarousal symptoms (Costanzo et al., 2016), and findings of decreased integrity have also been reported in patients suffering from anxiety disorders (Phan et al., 2009; Baur et al., 2011). Furthermore, integrity of the UNC plays a role in emotional empathy (Oishi et al., 2015). Relevance of enhanced UNC integrity for the reported improvement in anxiety symptoms (Hoge et al., 2013) and enhancement in empathic behaviors (Shapiro et al., 2011) following MBSR should therefore also be tested.

### Suggestions for Future Studies and Conclusion

The findings of the present study come with several limitations and therefore can only be taken as suggestive in nature. Future studies that investigate the impact of mindfulness practice on emotional learning and white matter plasticity should include larger sample sizes and employ an active control intervention (e.g., the Stress Management Education (Hoge et al., 2013) or Health Enhancement Program (MacCoon et al., 2012) that were specifically developed as active control interventions for MBSR to test mindfulness as an active ingredient). Future studies might also consider including clinical populations, such as patients suffering from anxiety disorders. If confirmed by future research, the findings described here might have great relevance for the better understanding of the mechanisms of mindfulness, and might potentially elucidate one way through which mindfulness exerts its beneficial effects on mental health and well-being.

## Author Contributions

Designed the study: BKH, TG, DNG, SWL, MRM. Performed the study: BKH, VB, TG, DNG, SWL, MRM. Analyzed the data: VB, BKH, TG, DNG, KK, CS, SWL. Prepared the manuscript: BKH, VB, TG, DNG, KK, CS, SWL, MRM.

## Funding

This work was supported by a Templeton Positive Neuroscience Award to BKH and MRM, a Mind and Life Varela Award to BKH, funding by the Kusala Foundation, Berlin to BKH, a Laura Bassi-Preis from the Technical University of Munich to BKH, and NIH award R01AT006344 to SWL. This research was carried out at the Athinoula A. Martinos Center for Biomedical Imaging at the Massachusetts General Hospital, using resources provided by the Center for Functional Neuroimaging Technologies, P41EB015896, a P41 Biotechnology Resource Grant supported by the National Institute of Biomedical Imaging and Bioengineering (NIBIB), National Institutes of Health. This work also involved the use of instrumentation supported by the NIH Shared Instrumentation Grant Program and/or High-End Instrumentation Grant Program; specifically, grant number(s) S10RR023401, and S10RR023043.

## Conflict of Interest Statement

The authors declare that the research was conducted in the absence of any commercial or financial relationships that could be construed as a potential conflict of interest.

## Footnotes

^1^https://redcap.partners.org/redcap/

^2^https://surfer.nmr.mgh.harvard.edu
